# Neurodevelopmental Outcomes of Pregnancies Resulting from Assisted Reproduction: A Review of the Literature

**DOI:** 10.3390/children9101511

**Published:** 2022-10-03

**Authors:** Paraskevas Perros, Alexandros Psarris, Panagiotis Antsaklis, Marianna Theodora, Michael Syndos, Antonios Koutras, Thomas Ntounis, Zaharias Fasoulakis, Alexandros Rodolakis, Georgios Daskalakis

**Affiliations:** 1st Department of Obstetrics and Gynecology, “Alexandra” General Hospital, National and Kapodistrian University of Athens, 11526 Athens, Greece

**Keywords:** assisted reproduction techniques (ART), neurodevelopment disorders, mental health, fertility treatments, assisted conception, natural conception (NC), in vitro fertilization (IVF), verbal ability, autism spectrum disorders (ASD), intracytoplasmic sperm injection (ICSI)

## Abstract

The term infertility is defined as the lack of conception within 1 year of unprotected intercourse. It affects more than 80 million individuals worldwide. It is estimated that 10-15% of couples of reproductive age are challenged by reproductive issues. Assisted reproduction techniques (ART) are responsible for more than 4% of live births. Our aim is to review the research on neurodevelopmental outcomes of newborns born after the implementation of assisted reproduction methods compared to those conceived naturally. We conducted a comprehensive search of the PubMed, Crossref and Google Scholar electronic databases for related articles up to June 2022 using the PRISMA guidelines. Our research revealed a large number of long term follow-up studies between 2 and 18 years of age, with comparable developmental outcomes. Many studies compared the effects of different infertility treatments against natural conception. The review of the literature revealed that ART is safe, as the majority of studies showed no effect on the neurodevelopmental outcomes of the offspring. In most cases when such an effect was observed, it could be attributed to confounding factors such as subfertility, multiple pregnancies and gestational age at delivery. Finally, the increase in the prevalence of neurodevelopmental disorders after ART, as described in studies with statistically significant results, is predominantly marginal, and given the low incidence of neurodevelopmental disorders in the general population, its clinical significance is debatable.

## 1. Introduction

The term infertility is defined as the lack of conception within 1 year of unprotected intercourse [1]. It affects more than 80 million individuals worldwide [1]. It is estimated that 10–15% of couples of reproductive age are challenged by reproductive issues [1]. These couples resort to assisted reproduction technology to achieve their reproductive goals. Assisted reproduction techniques (ART) are responsible for more than 4% of live births [2]. Concurrently, the number of oocyte donation (OD) treatment cycles performed every year in Europe and the US has reached 50,000 [3].

Assisted reproduction techniques have been associated with an increase in the prevalence of fetal morbidities such as increased blood pressure [4,5], metabolic disorders [6] and reproductive tract anomalies such as hypospadias [7]. Furthermore, it has been shown that among singleton pregnancies, adverse perinatal outcomes, including preterm delivery, placenta previa, low birth weight infants and a decreased incidence of spontaneous cephalic delivery are more common in individuals conceived with ART procedures regardless of the type of procedure used [8]. Multiple studies have suggested that the contribution of maternal factors associated with infertility to the adverse outcomes may be more important than the ART procedures themselves [8,9,10,11,12]. The results from multiple pregnancies are similar [13]. However, multiple gestations have been shown to have worse neonatal outcomes compared to singleton pregnancies [13].This has resulted in a move towards the transfer of a single embryo (SET), aiming to minimize the perinatal risks associated with children born after ART. 

When evaluating the effects of ART, we must keep in mind the heterogeneity of procedures utilized to treat infertility. Assisted reproduction techniques incorporate a variety of infertility treatments aiming to achieve conception such as artificial insemination, intrauterine insemination, ovulation induction, in vitro fertilization, intracytoplasmic sperm injection, cryopreservation of gametes and embryos and oocyte donation. It is evident that due to the great number of different procedures there is an increased number of possible associations to be evaluated. 

The aim of this review is to amass the research on the neurodevelopmental outcomes of newborns conceived with the use of assisted reproduction methods and compare it with newborns conceived naturally. Assisted reproduction techniques have been associated with an increase in the prevalence of fetal morbidities [4,5,6,7] and adverse perinatal outcomes. Hence, it is of the upmost importance to evaluate whether the neurodevelopmental outcomes of children born after ART are affected in comparison with children conceived naturally. In this review we will summarize all the latest data and evaluate the possible association of neurodevelopmental disorders with the different methods of assisted reproduction.

## 2. Materials and Methods

**Figure 1:**
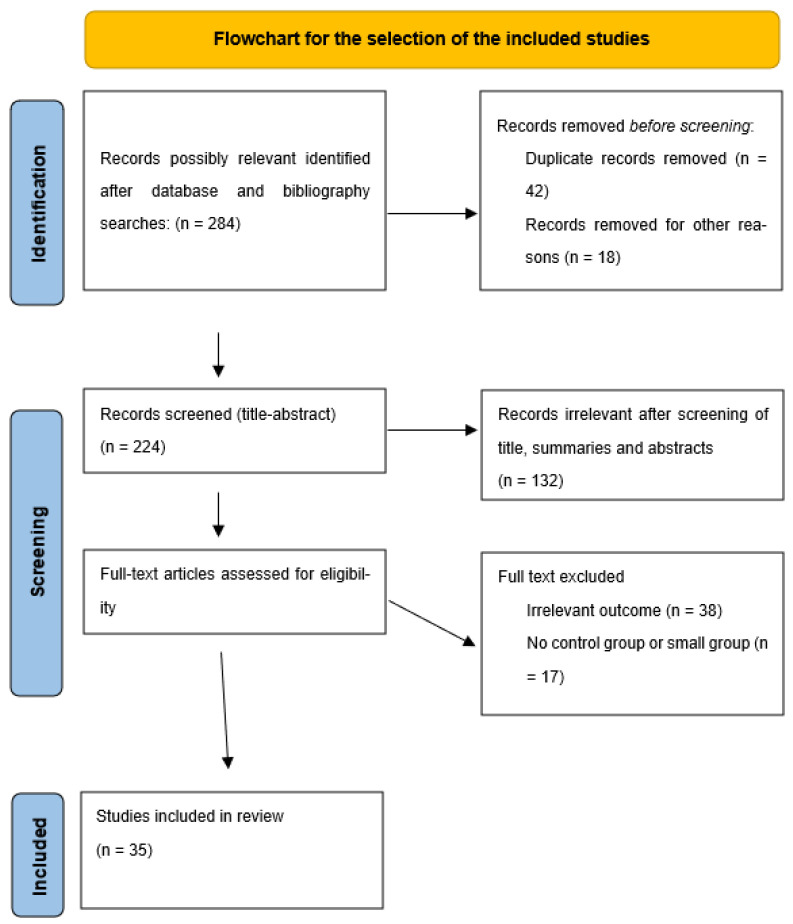
Flowchart of the selection of the included studies.

This review was planned, organized, and developed following the Preferred Reporting Items for Systematic Reviews and Meta-analyses (PRISMA) reporting guidelines (Figure 1) [14]. We conducted a comprehensive search of the PubMed, Crossref and Google Scholar electronic databases for articles up to June 2022, using the search terms assisted reproduction techniques (ART), neurodevelopment disorders, neurodevelopment outcomes, mental health, verbal ability, autism spectrum disorders (ASD), and cerebral palsy (CP) in conjunction with fertility treatments, assisted conception, natural conception (NC), in vitro fertilization (IVF), intracytoplasmic sperm injection (ICSI), frozen embryo and vitrified oocytes. Titles, summaries, and abstracts of all identified studies were checked for study design, type of association and outcome. The full text of relevant articles was carefully read and analyzed. Narrative and systematic reviews were excluded from our review, while cohort studies, case control studies and clinical trials were included in our study. 

## 3. Results

Given the variability of Assisted Reproduction Technologies used in current practice, we elected to present the results comparing the neurodevelopmental outcomes of ART vs. natural conception in general and then analyze specific aspects of ART such as intracytoplasmic sperm injection (ICSI), which has been the focus of a lot of research in the past few years. Furthermore, we compared the incidence of autism in offspring born via natural conception versus ART. All the studies analyzed below are also available in Table 1.

### 3.1. ART versus Natural Conception 

There are a lot of studies that have evaluated the neurodevelopmental outcomes of offspring conceived by ART vs. children conceived naturally. A very important prospective cohort study conducted by the Integrated Research Network in Perinatology of Quebec and Eastern Ontario in Canada compared cognitive, motor, and language neurodevelopmental outcomes between ART and natural conception groups at 24 months of age [17]. This study recruited 2366 pregnant women, of whom 278 conceived with ART between 2010 to 2012. This study, also known as the 3D-Study (Découvrir, Développer, Devenir), revealed no difference in cognitive scores, composite motor scores and language scores during the neurodevelopmental assessment at the age of 2 years old. Furthermore, no difference was observed when independent ART techniques were compared nor when comparing in vivo (ovarian stimulation or intrauterine insemination) or in vitro (in vitro fertilization, intracytoplasmic sperm injection, or in vitro maturation) techniques [17]. 

Another very important population-based, prospective cohort study was conducted using the Swedish national health archive [39]. This study included children born between 1982 and 2007 and followed for a clinical diagnosis of autistic disorder or mental retardation until the end of 2009 [39]. Out of more than 2.5 million infants born, 30.959 (1.2%) were conceived by ART, and they were followed up for a mean of ten years. There was no statistically significant association for either outcome after restricting analysis to singleton pregnancies [39]. 

Two studies from Denmark, with data from 1995 to 2003 and 2003 to 2008, respectively, compared IQ scores and selective or sustained attention scores between children conceived via ART and children born after natural conception [18,25]. These studies included 588,967 children (33,139 ART) and 1782 children (205 infertility group & 1577 spontaneously) respectively [18,25]. No statistically different outcomes were documented after correcting for confounding factors such as birthweight, maternal age and parity. 

Τwo smaller cohort studies evaluated the effects of ovarian stimulation on the neurodevelopmental outcomes of offspring. Τhe Groningen ART cohort study which included a total of 254 children (66 ovarian stimulation/IVF, 87 natural conception-subfertile and 101 reference group) and the Netherland prospective cohort study which included a total of 665 singletons (68 after controlled ovarian stimulation-IVF/ICSI, 57 natural cycle-IVF/ICSI, 90 naturally conceived singletons of subfertile couples and 450 control group) both concluded that the neurological outcome is not influenced by ovarian hyperstimulation [2,36]. 

The New York prospective cohort study included 5841 children (1830 ART & 2074 twins) through 36 months of age [46]. It analyzed five developmental domains (fine motor, gross motor, communication, personal-social functioning, and problem-solving ability), as measured by the parental completion of the Ages and Stages Questionnaires at 4, 8, 12, 18, 24, 30, and 36 months of age [46]. The study indicated that infertility treatment was not associated with an increased risk of the children failing in any developmental domain after excluding multiple deliveries [46]. 

Finally, a case control study from an Iranian Assisted Reproduction Center, which included 400 children conceived via ART and 420 controls conceived naturally, compared the neurodevelopmental status at 9 months old [28]. This study revealed no difference in the neurodevelopmental status at nine months between naturally conceived children and children born with ART.

It is apparent that in the absence of confounding factors, the literature overwhelmingly shows that the neurodevelopmental outcomes of children born after infertility treatment are on par with the neurodevelopmental outcomes of naturally conceived offspring.

### 3.2. Long-Term Follow Up

For the most part, studies that compare the neurodevelopmental outcomes of children born with ART vs. naturally conceived offspring focus in the first three years of life. However, given that several mental disorders are diagnosed later in life, it is important to assess the neurodevelopmental outcome of children during the first two decades of their life [20,47].

Zhu et al, utilized data from three population-based birth cohorts (the Aalborg-Odense Birth Cohort, the Aarhus Birth Cohort, and the Danish National Birth Cohort) from Denmark to compare the incidence of behavioral problems in children born to fertile and infertile couples [20]. The children studied were between the ages of 7 and 21 years. No statistically significant difference was detected regarding behavioral problems regardless of the presence of infertility and the infertility treatment used [20]. 

Another large population-based cohort study from the United Kingdom investigated the possible influence of infertility treatment to the cognitive development of offspring at the ages of 3 and 5 [42]. It included 18.818 children and concluded that neither subfertility nor ART adversely affected children’s cognitive development at ages 3 and 5 [42].

Similarly, other smaller studies observed no statistically significant differences in the neurological outcomes after long-term follow up of offspring conceived via ART vs. children conceived naturally. Takeshige et al. compared neurological outcomes of offspring conceived from vitrified oocytes after ICSI with the national average data from Japan at regular intervals between three months and six years of age [22]. No statistically significant differences were observed regarding the neurological outcomes [22]. Punamaki et al. prospectively followed up 255 singleton children born after ART (164 IVF/76 ICSI) and compared their cognitive development and mental health at the age of 7–8 years old with 278 naturally conceived children without detecting any statistically significant differences [9]. Wagenar et al. compared 139 adolescents born after IVF with 143 control adolescents regarding attention, information processing and visual-motor function, and did not detect any statistically significant differences [43]. A recent study from Farhi et al. compared different neurodevelopmental measures of children conceived by ART (*n* = 358) compared to spontaneously conceived offspring (*n* = 401), concluding that there is no statistically significant difference between the two groups [22]. Finally, Bay et al. conducted a prospective register-based cohort study in Denmark and included all the children born in Denmark between 1995 and 2003, with follow-up in 2012 when they were aged between 8 and 17 years old [19]. The numbers of the children studied were 33,139 conceived after fertility treatment and 555,828 after spontaneous conception. Conversely to the previously mentioned studies, the study from Denmark revealed a statistically significant increase in mental disorders in offspring born after ovulation induction/intrauterine insemination (iui) compared to naturally conceived offspring [19]. However, the same was not true about in vitro fertilization (IVF) and intracytoplasmic sperm injection (ICSI) in the same population [19]. No specific type of hormone drug treatment was related to the higher risk of mental disorders [19].

### 3.3. ICSI vs. Natural Conception 

The term intracytoplasmic sperm injection (ICSI) is used to describe the assisted reproductive method that involves choosing a single sperm cell and injecting it manually into the ovum [48]. 

There are a lot of studies evaluating the neurodevelopmental outcomes of children born after ICSI in comparison either with natural conception or other assisted reproduction techniques. The majority of both cohort [16,26,31,38,39,42,44] and case control studies [40,41] are in accordance with the notion that children conceived via ICSI are not at higher risk of mental disorders. 

One of the largest population-based cohort studies was conducted by the Taipei Medical University between 2004 and 2016 [44]. It included 23,885 children, (23,148 born after natural conception and 737 after ICSI) and concluded that there is no difference in the risk of developing neurodevelopmental disorders among the two groups [44]. On par with the previous study, a prospective cohort study conducted by Agarwal et al. compared the neurodevelopmental outcome at 2 years of age between 76 offspring conceived via ICSI and 261 matched controls, and concluded that neurodevelopmental and functional outcomes were similar in both groups [16]. 

A study conducted by Ponjaert-Kristoffersen et al. matched three hundred singleton children conceived via ICSI with spontaneously conceived controls from Belgium, Sweden, and the USA [38]. They compared their psychological well-being and cognitive development at the age of 5 and concluded that there was no statistically significant difference between the two groups [38]. A cohort study conducted in Australia included 89 children born after ICSI, 84 born after IVF and 80 conceived naturally [31]. The results of this study suggest that children conceived using ICSI do not have an increased risk of delayed mental development at 5 years of age [31]. 

Ludwig et al. conducted a prospective controlled single-blinded study to assess the neurodevelopmental health of children born after ICSI [33]. The study included 276 children born after ICSI and 273 naturally conceived singletons at 5.5 years old [33]. The results showed no difference regarding neurologic examination, motor skills, emotional/behavioral development, and intelligence [33]. Similarly, Sutcliffe et al. compared the outcomes of 208 children conceived via ICSI with 221 naturally conceived children at 17 months old. As with previously mentioned studies, no significant difference was detected regarding the neurodevelopmental ability of children conceived after ICSI vs. children conceived naturally [41]. A smaller study from the same lead author of a cohort of Australian children yielded the same results [40]. 

A small number of studies have associated ICSI with poorer neurodevelopmental outcomes of the offspring. However, most of these studies have included a very small number of patients and have neglected to account for confounding factors.

The study by Sandin et al. included a very large number of patients (2.5 million, 30.959 of whom born after ART) and followed them for a considerable amount of time (10 years) [39]. However, initial results showing an increase in the prevalence of mental retardation and autistic disorder in children born after ART were swiftly dismissed when restricting the analysis to singleton pregnancies [39]. As for the effects of ICSI on the development of offspring, after adjusting for singleton pregnancies, a statistically significantly association of ICSI with mental retardation was observed [39]. However, there are certain limitations one needs to consider in order to properly interpret these results. Firstly, the prevalence of mental retardation is very low, and the risk associated with the procedure is very small. Secondly, important confounding factors such as parental socioeconomic status were not evaluated during the analysis. Hence, the results of this study must be interpreted carefully. 

Knoester et al. conducted a study of singletons born between June and December 1996 at Leiden University Medical Center aged between 5 and 8 years old [29]. The researchers compared the IQ scores of 252 infants (83 born after ICSI, 83 born after IVF and 86 spontaneously conceived), and found that cognitive development was lower among singletons born after ICSI compared to the other two categories [29]. Although the results seem alarming, one has to consider the limited sample size, possible selection bias, unavailable parental IQ scores, and lack of clinical significance of the mean difference in IQ, which was between 3 and 7 points [29]. 

### 3.4. Autism Spectrum Disorders and ART

An aspect of the neurodevelopmental outcome of children which has come under a lot of attention in recent years is autism. Autism spectrum disorder (ASD) is a neurological and developmental disorder that affects the way people interact, communicate with others, learn, and behave [49]. It is important to investigate if offspring after ART are at an increased risk of autism spectrum disorders compared to those conceived naturally.

There are a few case-control studies that claim no association between autism spectrum disorders and ART. One study was conducted in Finland and studied 4164 cases with autism and 16,582 matched controls born in 1991–2005 [30]. Another study was conducted in Iran, and it included 100 cases with ASD and 200 controls between 2 and 10 years of age [27]. 

Larger studies, which reached the same conclusions, were conducted in Massachusetts, Taiwan, and California. The Massachusetts study included 10,147 children born after ART, 8072 offspring born from subfertile couples without the use of assisted reproduction and 441,898 children born from fertile couples and concluded that compared to children born to fertile women, children born to ART, ICSI, or IVF, or subfertile women are not at increased risk of receiving an ASD diagnosis [21]. The Taiwan Birth Cohort Study, using a national birth cohort dataset, reached the same conclusion regarding the lack of association between ART and ASD [34]. An observational cohort study from California studied all 5,926,251 live births from 1997 to 2007 and revealed no association between ART and ASD, while adjusting for possible confounding factors [23]. Another large population-based prospective cohort study using the Swedish national health registers from 1982 to 2007 showed no association between ART and ASD overall, while other possible associations between ASD and specific techniques such as ICSI were not statistically significant when the analysis was restricted to singleton pregnancies [39]. Finally, a prospective cohort study based on data from the Danish National Health Register which included all children born alive between 1995 and 2003 showed a comparable risk for ASD between children conceived naturally and children born after IVF or ICSI [19]. A marginal but statistically significant association was noted between induced ovulation and intrauterine insemination and ASD, which was not the case with any other study [19]. 

After reviewing all available research on the possible association between ART and ASD, the overwhelming majority of data support the absence of association. Hence, development of ASD should not be a concern for couples resorting to ART, as no association has been documented for the majority of ART techniques. However, since a possible mild association was noted in one of the published studies, further evaluation is warranted to determine the validity of the association between ovulation induction/iui and ASD. 

## 4. Discussion

This review summarizes the existing literature on the neurodevelopmental outcomes in offspring born after ART compared to those conceived naturally. Despite the initial perception that there is an association between ART and neurodevelopmental disorders, the elimination of confounding factors results in a lack of such an association in the majority of studies. 

When discussing neurodevelopmental outcomes, it is of great importance to analyze long-term outcomes. Several studies have looked into long-term neurodevelopmental outcomes of children born after ART. Long-term outcomes include comparisons of neurodevelopmental outcomes not only during early childhood but for the first two decades of their life [9,20,22,29,42,43,45,46]. None of the studies showed a greater risk of mental disorders for children born from ART, with the exception of a follow-up study from Denmark that showed a low but statistically significant risk of mental disorders after ovulation induction [19]. 

ICSI, being based on the non-natural selection of sperm, came under a lot of research regarding offspring outcomes. Recent data suggest that children born after ICSI do not have an increased risk of developing mental disorders [16,26,32,38,39,40,41,42,44]. However, two studies [38,44] observed a higher incidence of mental and psychological disorders in male offspring born after ICSI, and one study suggested a possible relationship between ICSI and mental retardation [39].

The majority of the studies that reported a possible association between ART and neurodevelopmental disorders in offspring did not take into account multiple gestation, maternal age, prematurity, birthweight, socioeconomic and health lifestyle differences. One of the most important contributors to poor neurodevelopmental outcomes is multiple gestation [23,25,35,45]. In many cases, limiting the analysis to singleton pregnancies diminishes any previously occurring statistically significant differences [45]. Furthermore, apart from multiple gestations, subfertility factors such as maternal age and paternal infertility have been associated with poorer neurodevelopmental outcomes in offspring [39]. Both Schendelaar et al. and Goldsmith et al. showed that poorer neurodevelopmental outcomes are associated with infertility rather than the assisted reproduction procedures [2,24]. Other factors highly associated with an increased prevalence of mental disorders include preterm delivery and low birth weight, [15,18,37,45]. Another weakness of the studies that show poorer neurodevelopmental outcomes after ART is the fact that the type of procedure linked to poorer neurodevelopmental outcomes varies between studies (ICSI, ovulation induction, intrauterine insemination etc.). Finally, the increase in the prevalence of neurodevelopmental disorders after ART, as described in studies with statistically significant results, is predominantly marginal, and given the low incidence of neurodevelopmental disorders in the general population, its clinical significance is debatable. 

On the other hand, one should not overlook the facts that some of the studies that have observed statistically significantly worse neurodevelopmental outcomes in children conceived after various ART procedures include a large number of patients [19,39]. These studies, despite their weaknesses, have been conducted with the proper methodology and have taken into account a number of confounding factors. It should be noted that adjusting the analysis for multiple confounding factors may be necessary, but it results in lowering the power of the study and hence results are less likely to be statistically significant. 

This review summarizes the existing literature on the potential relationship between assisted reproduction techniques and the risk of increased incidence of neurodevelopmental disorders in the offspring compared to pregnancies conceived naturally. Our study has some limitations. Firstly, great heterogeneity was observed among most of the analyzed studies. This is not surprising, as the included studies investigated diverse populations, from numerous geographic regions, different age groups, fertility treatments, ART procedures and delivery year (1984–2020). Consequently, the antenatal care varied depending on the country and year of delivery. However, it is important that the majority of the studies adjusted the statistical analysis for the most common confounding factors such as multiple gestations, birthweight, gestational age and maternal age. However, other confounding factors such as the socioeconomic status of the parents, mental health and educational level, which may affect the incidence of neurodevelopmental outcomes in the offspring, were not always considered. Finally, the term neurodevelopmental disorder is very diverse, including abnormalities ranging from mild disorders to very serious, debilitating conditions. Hence, comparison between different studies is even more challenging.

## 5. Conclusions

The greater part of the literature shows no association between ART procedures and poorer neurodevelopmental outcomes in offspring. However, there is a small number of studies that have demonstrated possible associations between various ART procedures and different neurodevelopmental disorders. The interpretation of these studies must be made carefully, as accounting for confounding factors may negate the proposed association. In any case, even when an association is proposed between ART and a worse neurodevelopmental outcome, the clinical impact is expected to be very small. Hence, ART procedures should be considered safe regarding the incidence of neurodevelopmental disorders in offspring.

## Figures and Tables

**Table 1:** Overview of studies on the neurodevelopmental outcomes of infants conceived via assisted reproduction techniques versus naturally conceived children.

Study	Country/Region	Design	Duration	Assisted Conception	Sample Size		Neurodevelopmental Outcomes	Follow-Up (Years)	Results	Cofounders
					Art	Control Group				
Abdel-Latif et al., 2013 [15]	New South Wale Australian Capital Territory	Population-based retrospective cohort study	1998–2004	IVF	217	1256	Developmental Delay Cerebral palsy Deafness Blindness	2–3	Mortality & Age no difference Higher rate of functional disability singletons born by IVF born at 22-26w (aOR 1.79, 95% CI 1.05 to 3.05, *p* = 0.03) but not at 27–28w (aOR 0.81, 95% CI 0.37 to 1.77; *p* = 0.59) than those after SC.	Gestational Age Multiple birth
Agarwal et al., 2005 [16]	Tertiary care perinatal centre	Prospective cohort study	13 months	ICSI	76	261	Prospective register based cohort study	2	No risk	Maternal age Sex Gestational age parity
Balayla et al., 2017 [17]	Integrated Research Network in perinatology of Quebec and Eastern Ontario (Canada)	Prospective Cohort Study	2010–2012	ALL TECHNIQUES Stimulation (*n* = 53) UI (*n* = 79) IVF (*n* = 32) ICSI (*n* = 105) In vitro maturation (*n* = 9)	278	2088	Cognitive scores Motor scores Language score	0–2	No significant difference (*p* > 0.05)	
Bay et al., 2014 [18]	Denmark	Follow-up	2003–2008	All	205	1577	Neurodeve-lopmental assessment	0–5	Intelligence (md: 0.6 95% CI: 2.2–3.4) Overall attention (0.1, 95% CI: 0.2–0.4)	
Bay et al., 2013 [19]	Denmark	Prospective Register Based Cohort study	1995–2003	All	33139	555828	Mental disorders	8–17	Statistically significant increase in mental disorders after ovulation induction (1.20, 1.11 to 1.31;absolute risk 4.1%)	Maternal age Smoke Psychiatric history Educational level
Carson et al., 2011 [20]	United Kingdom	Prospective population based cohort study	2000–2002	All Techniques	ART (*n* = 96) Ιnduced Ovulation (I = 167)	11873	Cognitive development-verbal ability	3 & 5	No significant difference	Sociodemographic Factors Multiple pregnancy
Diop et al., 2019 [21]	Massachusetts Taiwan California	longitudinal cohort study	2004–2013	All	10147 (ART) 8072 (subfertile)	441898	ASD	0–3	No significant difference (*p* < 0.05)	Social life Smoke Maternal age Prenatal care Chronic hypertension diabetes
Farhi et al., 2021 [22]	Israel	Follow-up Study		All	358	401	Developmental coordinationShort Sensory profileAutismAttention-deficit hyperactive disorder	7–8	No significant difference	
Fountain et al., 2015 [23]	California	Observation cohort study	1997–2007	All	48865 (ART) 32922 (IVF)	59262251	ASD		No increased risk for ASD after ART	Demographic Adverse prenatal & perinatal outcomes
Goldsmith et al., 2018 [24]	Western Australia	Cohort study	1994–2002	All	2914	208746	Cerebral pulsy	0–5	No significant difference	Gestational Age Multiple birth
Hvidtjørn et al., 2010 [25]	Denmark	Population based follow-up	1995–2003	Ivf (14991—2.5%) OΙ (18148—3.1%)	33.139 (5.6%)	588.967	Cerebral palsy	5–13	HRR 1.45 (95% CI: 0.96–2.19).	Multiple Birth Maternal Age Parity Birthweight Smoke
Husen et al., 2021 [26]	Rotterdam	Cohort Study		IVFICSI	50	116	Embryonic brain development	9w & 11w	9w: no difference 11w: slightly larger at ART embryos	Multiple Births Smoke Gestational AgeLow Birth weightPreterm BirthHypertensive DisordersCongenital Anomalies
Jenabi et al., 2020 [27]	IRAN	Case control study		AlL	100 (ASD)	200	ASD	2–10	No significant association between IVF and ASDs (OR): 0.9, 95% (CI): 0.7–1.3	Sex History of preterm birth Maternal age
Kermani et al., 2011 [28]	Iranian Assisted reproduction center	Case control		All Techniques	400	420	Developmental Assessment	0–9 months	No significant difference (*p* > 0.05)	Premature Multiple pregnancy
Knoester et al., 2008 [29]	Leiden university medical center	Follow-up	1996–1999	ICSI	83 ICSI 83 IVF	86	IQ score	5–8	Lower at Art	Not concerned: Small sample Parents IQ
Lehti et al., 2013 [30]	Finland	Control study	1991–2005	ALL	4164 (autistic)	16582	ASD	0–16	No significant association was found between IVF and ASDs (OR: 0.9, 95%), (CI: 0.7–1.3)	
Leslie et al., 2003 [31]	Australian	Cohort study		ICSI IVF	84 IVF 89 ICI	80	Developmental disorders	1 & 5	No significant difference	Gestational age Twins Educational level
Leunens et al., 2008 [32]	Belgium	Follow-Up		ICSI	109	90	Cognitive Abilities Motor Development	0–10	No risk	Maternal Age Need of ICU
Ludwig et al., 2009 [33]	Tertiary care perinatal centre	Prospective control single-blinded		ICSI	276	273	Neurodevelopmental health (motor skills, emotional behavioral development, intelligence)	5.5	No significant difference (*p* < 0.05)	
Lung et al., 2018 [34]	Taiwan	Cohort study		ALL	744 (ART) 415 (ASD)	20095	ASD	0–5.5	No increased risk for ASD after ART	
Maheshwari et al., 2016 [35]	UK	Retrospective Cohort Study	1991–2011	Fresh vs. Frozen embryo (IVF, ICSI)	16521	95911	Birth weight	0	no difference in RR of preterm birth (0.96 (0.88–1.03)), very preterm birth (0.86 (0.70–1.05)), and congenital anomalies (0.86 (0.73–1.01)) RR of having a high birth weight baby was higher (1.64 (1.53–1.76)) on frozen	Age Parity Year of treatment Duration of treatment
Middelburg et al., 2009 [36]	Groningen	Prospective cohort study	3/2005-12/2006	Ovarian Hyperstimulation IVF ICSI	Ovarian hyper/tion (*n* = 68) Natural cycle (I = 57)	90	Neurological Condition	4, 10 & 18 months	No significant difference	Gestational Age Demographic Factors Maternal Age
Middelburg et al., 2010 [37]	Netherland University Medical Center Groningen	Prospective Cohort Study	3/2005-12/2006	IVF ICSI	Ovarian hyperstimulation (*n* = 68) Natural cycle (*n* = 57)	540	Neuromotor Development	0–3 months	No significant difference	Gestational Age Demographic Factors Maternal Age
Place & Englert, 2003 [12]	Brussels	Prospective longitudinal study	4/1998-3/2020	ICSI IVF	ICSI (*n* = 66) IVF (*n* = 52)	59	Psychomotor Intellectual Development	0–5	No significant difference	Gestational Age Birth Weight
Ponjaert-Kristoffersen et al., 2004 [38]	Belgium Sweden USA	Multicentre control study		ICSI	300	260	Psychological outcomes cognitive abilities	0–5	No significant difference (*p* < 0.05)	Gender Maternal age Gestational age
Punamäki et al., 2016 [9]	Finland	Prospective Follow-up study		IVF ICSI	164 IVF 76 ICSI	278	Mental health Social Cognitive abilities	7–8	No significant difference	Father’s age Mother’s parity Gestational age Need of Intensive Care Unit
Sandin et al., 2013 [39]	Swedish National Health Archive	Prospective Cohort Study	1982–2007	All techniques	30,959 (1.2%)	2.5 M	Autism Mental retardation	0–10	No difference	Multiple Birth
Schendelaar et al., 2011 [2]	Groningen	Cohort study		IVF (Hyper/tion & Natural cycle)	Hyper/tion IVF (*n* = 66) Natural cycle IVF (*n* = 56) Subfertile-no IVF (*n* = 87)	101	Neurodeve-lopmental assessment	0–2	No significant difference	Perinatal outcomes Social factors
Sutcliffe et al., 2003 [40]	Austalia vs. UK	Retrospective case-control study		ICSI	58 & 208 (UK)	38 & 221 (UK)	Neurodevelopmental disorders perinatal outcomes congenital abilities	15 months	No significant difference (*p* < 0.05)	Maternal age Sex Social class
Sutcliffe et al., 2001 [41]	UK	Case-Control study		ICSI	208	221	Neurodevelopmental disorders perinatal outcomes congenital abilities	17 months	No significant difference (*p* < 0.05)	Maternal age Sex Social class
Takeshige et al., 2021 [42]	Japan	Follow-up	2000–2020	ICSI	116	All born (national average)	Mental & Physical development	0–6	No significant difference	
Wagenaar et al., 2009 [43]	German	Case control study		All	139	143	Attention Visual-motor function	9–18(mean: 13.5)	No significant difference	
Wang et al., 2021 [44]	Taipei Medical University	Population-based cohort study	2004–2016	ICSI	737	23148	Neurodevelopmental disorders	3–5	No Risk	Male sex Intensive care unit admission
Yeung et al., 2016 [45]	New York State	Prospective Cohort Study	2008–2010	IVF Ovarian induction Intrauterine insemination	1830	4011	Fine motor Gross motor Communication Personal-social functionality Problem-Solving Ability	0–3	No significant difference (aOR, 1.33; 95% CI, 0.94–1.89)	Multiple birth Birth weight
Zhu et al., 2011 [46]	Aalborg-Odense, Aarhus, Danish National	Cohort study	1984–1987 1990–1992 1996–2002	all			Behavioral problems	7–21	No significant difference	

ART: assisted reproduction techniques; IVF: in-vitro fertilization; UI: uterine insemination; ICSI: Intracytoplasmic sperm injection; ASD: autism spectrum disease; NC: natural conception; SC: subsequent conception; IQ: intelligence quotient; OR: odds ratio; aOR: advanced odds ratio; CI: confidence interval; RR: relative risk; ΙCU: intensive care unit.

## Data Availability

Not applicable.
